# Anti-Ri antibodies associated with short-term memory deficits and a mature cystic teratoma of the ovary

**DOI:** 10.1186/1477-7800-1-11

**Published:** 2004-11-10

**Authors:** Oluwole Fadare, Hassan J Hart

**Affiliations:** 1 Department of Pathology, Yale University School of Medicine, New Haven, CT, USA; 2 Neurology Central Associates, Stamford, CT, USA

## Abstract

**Background:**

The IgG autoantibody ANNA-2 (anti-Ri) is a type 2 antineuronal antibody that has been found to bind to highly conserved and widely distributed adult brain proteins encoded by the *Nova-1 *and *Nova-2 *genes. Anti-Ri antibodies are typically detected in the serum and cerebrospinal fluids of patients with neurological disorders such as opsoclonus/myoclonus and cerebellar ataxia and in association with gynecologic and breast malignancies.

**Case Presentation:**

This report describes an unusual example of a 33-year-old female patient who developed short-term memory deficits over a 3-month period. An extensive neurological work-up, including a panel of paraneoplastic markers was negative with the exception of a high titer serum Anti-Ri (1:15,3600). A large left ovarian mass was palpated, surgically resected and eventually diagnosed as a mature cystic teratoma. Post-operatively, memory deficits had disappeared within 1 month and serum Anti-Ri titers had decreased significantly to 1:256. An extensive diagnostic work-up for other malignancies was negative.

**Conclusion:**

Although, Anti-Ri antibodies are typically associated with malignancies, this case illustrates the potential association between benign tumors and this autoantibody.

## Background

Paraneoplastic syndromes (PNS) are signs or symptoms attributable to tissue damage at sites that are remote from a primary malignancy or their metastases. PNS involving virtually every level of the neuro-muscular system have been described [1-3]. Typically, the paraneoplastic syndromes causing neurologic disorders precede the diagnosis of the neoplasm and are often the main reasons that medical attention is sought [2,3]. Recent attention has thus centered on discovering novel serum or cerebrospinal fluid markers that can specifically identify not only the presence of a malignancy, but the type of malignancy involved if present.

A wide variety of anti-neuronal antibodies have been associated with the many PNS-associated neurologic disorders [2]. These antibodies have varying degrees of sensitivity and specificity for the underlying types of malignancies. However, in the right setting, the presence of a given auto-antibody in combination with specific signs and symptoms, may reasonably predict the primary site and tumor type. The vast majority of tumors associated with PNS-neurological disorders are malignant [2]. Notable exceptions include the identification of antibodies to voltage gated potassium channels (VGKC) in patients with thymomas [4]. We describe in this report the finding of a common anti-neuronal antibody (anti-Ri) in a patient with a benign neoplasm: mature cystic teratoma of the ovary, and whose neurologic symptoms, short-term memory deficits, was apparently associated with her tumor.

## Clinical history

A 33-year-old nulligravid female with no significant past medical history presented to her physician with complaints of a short-term memory loss of approximately three months' duration. This included an inability to remember details about 24-hour old events. There had been no major socio-economic or personal changes in her life over this period. A detailed neurologic examination was notable only for her presenting complaint. Routine laboratory work-up, including a lumbar puncture were all within normal limits. A physical examination revealed a large right adnexal mass, which upon ultrasonographic assessment showed internal features suggestive of a malignancy. A panel of serum paraneoplastic autoantibodies was then requested, including anti-Hu, anti-Yo, anti-Ri, anti-Tr and anti-Ma1/2. All were normal with the exception of IgG anti-Ri, measured at 1:15,360 by an indirect immunofluorescence method. An extensive diagnostic work-up failed to reveal any malignancies. The patient subsequently underwent a right salpingo-oophorectomy, and her adnexal mass was diagnosed as a benign mature cystic teratoma of the ovary. Almost immediately following her surgery, the patient expressed a subjective improvement in her symptoms. Within a month, the serum anti-Ri had decreased to 1:256, and a detailed neurologic examination revealed resolution of her symptoms. She has not experienced any relapse in her symptoms in the 1 year since her surgery.

## Discussion

In 1988 and 1991, Budde-Steffen at al [5] and Luque et al [6] described a subpopulation of patients with opsoclonus and a history of breast cancer in whose serum and CSF were identified an antibody that reacted against 55 kD and 80 kD proteins that were designated Anti-Ri (also known as ANNA-2). It has since been shown that these antigens are highly conserved but widely distributed central nervous system neuronal proteins which are encoded by the *Nova-1 *and *Nova-2 *genes and which may play a role in neuronal maturation and homeostasis [7-9]. As those seminal reports indicate, Anti-Ri was initially associated with opsoclonus/myoclonus and cerebellar ataxia symptomatology in patients with breast and gynecologic cancers. They have however subsequently been associated with a wide variety of malignancies that have included lung, gastric and bladder carcinomas [10]. Indeed, in one study, 32% and 36% of cancers associated with anti-Ri in patients with suspected PNS were breast and lung carcinomas respectively [10]. The spectrum of associated neurologic symptoms has also expanded considerably and now includes vertigo, muscle weakness, dysarthria, dysphagia, dementia, deafness, myelopathy, opthalmoplegia, encephalomyelitis, rigidity, nausea, myelopathy, sensorimotor neuropathy [10]. What these cases illustrate is that with a few exceptions [4], the vast majority of tumors associated with PNS-neurological disorders are malignant. Our case thus highlights the potential association between a benign neoplasm and the presence of these antibodies and neuronal symptomatology.

Is it possible that the presence of the autoantibody and the ovarian tumor are completely fortuitous?. Given the temporal relationship between the resolution of her symptoms and the sharp decrease in her anti-Ri titer following her surgery, we believe this is unlikely. However, the precise mechanistic basis for this association as well as potential influence of outside factors remains to be elucidated. It should also be noted that high titers of Anti-Ri have been identified in patients with a history of ovarian cancer but without any evidence of a PNS, suggesting caution in assessing the specificity of this auto-antibody for PNS.

In summary, we describe in this report an association of a common anti-neuronal antibody (anti-Ri) in a patient with a benign neoplasm, mature cystic teratoma of the ovary, and whose neurologic symptoms, short-term memory deficits, was apparently associated with her tumor. Although, Anti-Ri antibodies are typically associated with malignancies, this case illustrates the potential association between benign tumors and this autoantibody.

## Competing interests

The authors declare that they have no competing interests.

## Authors' contributions

Dr Fadare wrote the initial version of the manuscript.

Dr Hart managed the patient, provided clinical information, and revised the manuscript.
